# Multimodal Evidence of Atypical Processing of Eye Gaze and Facial Emotion in Children With Autistic Traits

**DOI:** 10.3389/fnhum.2022.733852

**Published:** 2022-02-15

**Authors:** Shadi Bagherzadeh-Azbari, Gilbert Ka Bo Lau, Guang Ouyang, Changsong Zhou, Andrea Hildebrandt, Werner Sommer, Ming Lui

**Affiliations:** ^1^Department of Psychology, Humboldt-Universität zu Berlin, Berlin, Germany; ^2^Center for Child Development, Hong Kong Baptist University, Kowloon Tong, Hong Kong SAR, China; ^3^Faculty of Education, The University of Hong Kong, Pokfulam, Hong Kong SAR, China; ^4^Department of Physics, Centre for Nonlinear Studies, Hong Kong Baptist University, Kowloon Tong, Hong Kong SAR, China; ^5^Beijing-Hong Kong-Singapore Joint Centre for Nonlinear and Complex Systems, Institute of Computational and Theoretical Studies, Hong Kong Baptist University, Kowloon Tong, Hong Kong SAR, China; ^6^Department of Psychology and Research Center Neurosensory Science, Carl von Ossietzky Universität Oldenburg, Oldenburg, Germany; ^7^Department of Psychology, Zhejiang Normal University, Jinhua, China; ^8^Department of Education Studies, Hong Kong Baptist University, Kowloon Tong, Hong Kong SAR, China

**Keywords:** gaze direction, emotion processing, face recognition, N170, EPN, autism spectrum disorder, ADOS

## Abstract

According to the shared signal hypothesis (SSH) the impact of facial expressions on emotion processing partially depends on whether the gaze is directed toward or away from the observer. In autism spectrum disorder (ASD) several aspects of face processing have been found to be atypical, including attention to eye gaze and the identification of emotional expressions. However, there is little research on how gaze direction affects emotional expression processing in typically developing (TD) individuals and in those with ASD. This question is investigated here in two multimodal experiments. Experiment 1 required processing eye gaze direction while faces differed in emotional expression. Forty-seven children (aged 9–12 years) participated. Their Autism Diagnostic Observation Schedule (ADOS) scores ranged from 0 to 6 in the experiment. Event-related potentials (ERPs) were sensitive to gaze direction and emotion, but emotion processing did not depend on gaze direction. However, for angry faces the gaze direction effect on the N170 amplitude, as typically observed in TD individuals, diminished with increasing ADOS score. For neutral expressions this correlation was not significant. Experiment 2 required explicit emotion classifications in a facial emotion composite task while eye gaze was manipulated incidentally. A group of 22 children with ASD was compared to a propensity score-matched group of TD children (mean age = 13 years). The same comparison was carried out for a subgroup of nine children with ASD who were less trained in social cognition, according to clinician’s report. The ASD group performed overall worse in emotion recognition than the TD group, independently of emotion or gaze direction. However, for disgust expressions, eye tracking data revealed that TD children fixated relatively longer on the eyes of the stimulus face with a direct gaze as compared with averted gaze. In children with ASD we observed no such modulation of fixation behavior as a function of gaze direction. Overall, the present findings from ERPs and eye tracking confirm the hypothesis of an impaired sensitivity to gaze direction in children with ASD or elevated autistic traits, at least for specific emotions. Therefore, we conclude that multimodal investigations of the interaction between emotional processing and stimulus gaze direction are promising to understand the characteristics of individuals differing along the autism trait dimension.

## Introduction

Impairments in social, emotional and communicative abilities are core symptoms of autism spectrum disorder (ASD; American Psychiatric Association, 2013). These abilities are closely related to eye gaze and facial emotional expression processing (Adams and Kleck, 2003). Many studies have shown that gaze processing deficits in autism may be due to impairments in using eye gaze as a proxy to understand facial expressions, intentions, and mental states of others (Baron-Cohen, 1995; Baron-Cohen et al., 1997, 2001; Leekam et al., 2000). The struggle to recognize emotions from facial expressions is one of the earliest identifiable markers of ASD (Dawson et al., 2005). In a large sample Reed et al. (2020) have found behavioral and genetic evidence for poorer emotion recognition with increasing autistic traits. In neuroimaging studies using facial emotion recognition tasks (Harms et al., 2010), individuals with ASD demonstrated altered processing (Johnson et al., 2015) in the amygdala (Dalton et al., 2005), fusiform gyri (Pierce et al., 2004; Pierce and Redcay, 2008), and posterior superior temporal gyri (Pelphrey et al., 2005). On the behavioral level, individuals with ASD have shown altered emotion recognition of positive and negative facial expressions with larger impairments in processing fear, anger, sadness, and disgust emotions as compared to happy emotions (Wong et al., 2008). However, in some previous studies there were no performance differences between individuals with ASD and typically developing (TD) children in facial emotion recognition tasks (Castelli, 2005; Jones et al., 2011; Fink et al., 2014).

### Event-Related Potential Studies on Face and Eye Gaze Processing in Autism Spectrum Disorder

Event-related potential (ERP) studies indicate difficulties of individuals with ASD in orienting to social stimuli. This was demonstrated by a reduced or delayed N170 response to faces, which may indicate impaired structural processing of faces (Samaey et al., 2020) or diminished emotion recognition (Chronaki, 2016). The N170 is one of the most frequently investigated face-sensitive ERP components, and is also associated with eye gaze processing (Pelphrey et al., 2005; Senju et al., 2005b; Webb et al., 2006). The N170 is therefore of great interest for investigating altered face processing in autism [for reviews see Monteiro et al. (2017) and Kang et al. (2018)]. In individuals with ASD, as compared to TD, longer N170 latencies to faces and smaller amplitudes to emotional facial stimuli have been found (de Jong et al., 2008; Batty et al., 2011; Tye et al., 2014). For example, Webb et al. (2006) reported longer N170 latencies to faces in children with ASD as compared with TD individuals, indicating a deviant pattern of brain responses to faces at an early age. With respect to specific emotions, previous studies demonstrated stronger increases of N170 amplitudes to fearful over neutral expressions in a control group as compared to an ASD group; in contrast, the N170 amplitudes to neutral faces did not significantly differ between these groups (de Jong et al., 2008; Faja et al., 2016). Wagner et al. (2013) and Faja et al. (2016) reported increased N170 amplitudes to happy and angry faces, only for a TD group but not for an ASD group. However, Tye et al. (2014) found larger N170 amplitudes for neutral as compared to fearful expressions only in ASD participants.

Evidence of unusual eye gaze direction processing among children with ASD was found in two ERP studies. Grice et al. (2005) recorded high-density ERPs from children (aged 3.5–7 years) with ASD while passively viewing faces with different gaze directions. The occipito-parietal negativity was larger in a direct than an averted gaze condition in children with ASD, resembling data collected from 4 months-old infants (Farroni et al., 2002). In contrast, ERPs of age-matched TD children and adults were not sensitive to perceived gaze direction (Grice et al., 2005), suggesting a developmental delay in the ASD group. The absence of gaze direction effects in TD individuals reported by Grice et al. (2005) is surprising, given the sensitivity to perceived eye gaze direction in other ERP studies. This is also at variance with findings of Senju et al. (2005a) who investigated ERP correlates in an active gaze direction detection task in children with ASD and TD children (*M* = 12 years). N170 to direct gaze was larger than to averted gaze in controls but not in the ASD group. After gaze direction changes, the N170 was followed by an enhanced occipito-temporal negativity (N2), which was lateralized to the right hemisphere and larger for direct than averted gaze for TD children but not for children with ASD. Similar problems with gaze processing have been reported on the performance level, unlike children with ASD, TD children showed an advantage in detecting direct gaze over averted gaze (Senju et al., 2005a; Senju and Johnson, 2009).

A later ERP component, the early posterior negativity (EPN) is considered to indicate reflexive visual attention to emotional stimuli, facilitating sensory encoding. Thus, both negative and positive emotional stimuli enhance EPN amplitudes as compared to neutral stimuli (Schupp et al., 2003; Foti et al., 2009; Holmes et al., 2009; Schacht and Sommer, 2009). A study found that adults with ASD had different hemispheric distribution of EPN in response to facial expression, as compared to neurotypical adults (e.g., Faja et al., 2016). Faja et al. (2016) found that adults with ASD differed from neurotypical participants by showing a reduced sensitivity to emotional information in the EPN but not in the preceding P1 or N170 components. The authors concluded that the N170, which is associated with perceiving information that is needed to distinguish faces from other object categories (Bentin et al., 1996), is not modulated differentially by emotional expressions in adults with ASD relative to neurotypical adults. All in all, a diminished EPN in adults with ASD suggests that emotional cues are perceived or attended less than in normotypical individuals. However, to the best of our knowledge, there are no such studies on children with a diagnosis of autism or high on autistic traits. It remains to be seen, however, whether this is also the case in children with high autistic traits.

Interactive aspects of facial emotion expression perception and eye gaze processing are often emphasized as crucial issues in autism (Grice et al., 2005; Senju et al., 2005b; de Jong et al., 2008; Akechi et al., 2010; Tye et al., 2013). Akechi et al. (2010) investigated the neural correlates of processing facial expressions with different gaze directions. Approach-oriented expressions (e.g., anger) combined with direct gaze elicited a larger N170 than avoidance-oriented expressions (e.g., fear) combined with averted gaze in TD children but less so in the ASD group. This finding suggests that gaze direction modulates the effect of emotional facial expressions. In an attention cueing task, de Jong et al. (2008) presented fearful and neutral faces with different gaze directions either in static and dynamic conditions. Children with ASD processed gaze cues typically when static neutral faces were presented, exhibiting larger N200 amplitudes and shorter RTs in validly cued conditions. However, in the dynamic condition, attention orienting was influenced by emotion only in the control group but not in the ASD group. These effects were taken to suggest an impairment of processing social information in individuals with ASD. Emotional expression and gaze direction interact, and jointly contribute to approach- or avoidance-related basic behavioral motivations.

The interaction of face and eye gaze processing is in line with the “shared signal hypothesis” (SSH; Adams and Kleck, 2003), which postulates that when gaze direction matches the intent communicated by a specific expression, it enhances the perception of that emotion. For example, happy and angry expressions are both categorized as “approach-oriented emotions,” and hence are usually better recognized in faces that look directly at the observer. In contrast, disgusted and sad expressions are categorized as “avoidance-oriented emotions,” and are more easily recognized when accompanied by an averted gaze. Importantly, it is suggested that children with ASD have difficulties in recognizing other’s facial expressions, especially anger (Bal et al., 2010). It is therefore of great interest to study, whether autistic individuals can benefit from this interaction of emotional expression and gaze direction in the same way as normal controls do, and to see if the SSH relates to other concepts about how ASD individuals processes facial expressions and eye gaze. For example, the “eye avoidance hypothesis” proposes that atypical gaze behavior in autistic individuals is due to a lack of social interest (Tanaka and Sung, 2016). Tanaka and Sung (2016) consider avoidance of the eye region as an adaptive strategy for autistic individuals, as they often perceive eye gaze as socially threatening and unpleasant. However, avoiding the eyes severely limits the possibility of recognizing a person’s identity, emotional expression and intentions from his/her face. Tanaka and Sung (2016) believed that this avoidance behavior is the most plausible explanation for the autistic deficits found so far. To investigate such interaction strategies, methodologies such as eye-tracking provide valuable behavioral measures of individuals with ASD.

### Eye-Tracking Studies on Face and Eye Gaze Processing in Autism Spectrum Disorder

Eye-tracking technology has been adopted in autism research for studying atypical gaze fixation on primary facial regions, such as the eyes. Chita-Tegmark (2016) conducted a meta-analysis of 68 studies on the allocation of attention in autistic individuals, inferred from fixation durations on faces, specific face regions (eyes, mouth), the body and non-social stimulus elements. The findings confirmed the commonly assumed atypical gaze patterns in autism. Across all studies, gaze times on the eyes, mouth, and face were reduced in autistic individuals as they looked more at the body and less at social details. According to the author, although effect sizes are small, gaze behaviors of autistic individuals consistently differ from healthy controls (also see Papagiannopoulou et al., 2014).

The findings on eye avoidance, a critical feature of face perception in individuals with ASD, suggest that recognition of basic emotions in autism is deficient, especially when the eye region is relevant. Individuals with ASD are less able to understand the “language of the eyes” and often cannot clearly assign subtle information from eye signals (Baron-Cohen et al., 1997). Song et al. (2012) examined the ability to recognize emotions in autistic children aged 6–12 with regard to looking at eye regions. They observed that it was easier for autistic children to look into another person’s eyes while processing positive emotions than negative emotions. In emotion recognition for happiness, autistic individuals were able to assess facial expressions using the eye region as competently as TDs. This seems to contradict the “eye avoidance hypothesis.” However, the authors suggested that atypical gaze behavior in autism is more likely to result in recognition of negative emotions, such as an angry facial. Later, Song et al. (2016) found that autistic individuals show a remarkable reduction in the processing of the eye region and an increased processing of the mouth region in fearful faces, when compared to their TD group. The authors suggested that autistic individuals may look less in the eyes of fearful faces because they experience a higher level of arousal, making them feel uncomfortable.

Support for differences in gaze behavior and its influence on the ability of autistic individuals to recognize emotions is not universal. Thus, Hernandez et al. (2009) examined the gaze behavior of autistic and healthy adults during the exploration of neutral and emotional facial expressions by means of eye tracking. In contrast to previous work assuming that individuals with ASD show a general disinterest in the eye region, both autistic and TD adults looked more frequently at the eye region than at other areas of the face. However, this study with only 11 adults with ASD was low powered. In an emotion 1-back task, Leung et al. (2013) studied fixation behavior in autistic children and TD children when they looked at pictures of disintegrated faces (with eyes separated) and normal faces. The results showed no difference between the groups with regard to the ability to recognize emotions and the number of fixations. Since both groups fixated the eyes more often and performed better when the eyes were presented together, the authors argued that also for individuals with ASD the eyes are the most important source of information during emotion recognition. However, since the autistic group showed increased fixation durations, recognizing emotions from the eyes may have been more effortful for them. Matsuda et al. (2015) also failed to find group differences between children with ASD and TD children in their fixation behavior at static emotional facial expressions (including surprise, happiness, anger, and sadness). Participants in both groups fixated longer on the eye regions of angry and sad than surprised faces but fixated longer on the mouth region in surprised and happy than angry and sad faces. According to the authors, this complements prior findings, showing the key role of the eye region in recognizing angry and sad expressions, and the importance of the mouth region for the recognition of surprised and happy faces.

Together, the findings on the influence of gaze behavior on facial perception and emotion recognition from facial expressions are inconsistent. Atypical gaze patterns seem to be generally well documented for autistic children and adolescents (Papagiannopoulou et al., 2014) but the effects of these differences and their manifestations in the preference or avoidance of certain facial regions are still unclear. However, the atypical avoidance of the eye region could explain autistic deficits regarding the processing of fear expressions (Lozier et al., 2014; Tell et al., 2014). In the few existing studies on the ability to recognize emotions from facial expressions, priority was given to facial stimuli of adults for selected emotions. As a result, there is a lack of research into the relationships of processing facial expressions and gaze perception and eye movements in autism, especially in children.

Recently, the Research Domain Criteria (RDoC) approach advocates a shift from treating mental disorders as categories to examining the continuum of symptom severity and diversity spanning the entire population (Insel et al., 2010; Cuthbert, 2015). In line with this approach, a growing body of studies investigated autism-associated social, emotional and communicative traits in the population, involving a broad range of individuals within or outside the autism spectrum (Abu-Akel et al., 2019). In line with RDoC, adaptive and maladaptive traits need to be characterized from a multimodal perspective, involving neural correlates and behavioral manifestations. Therefore, describing behavioral and neural correlates and associations of facial expression processing and their interactions with gaze direction and how they relate with continuous autism traits and clinical manifestations may contribute to better understanding of autism at a mechanistic level. The results of such an approach have the potential to explain hitherto reported mixed findings in neuro-typical and clinical populations.

Toward these aims, we report two experiments investigating the interactions between facial expressions and gaze direction and their relationship to social, emotional and communicative impairments in children with different degrees of autistic trait expressions. Experiment 1 recorded ERPs in response to angry and neutral facial expressions in children with varying degrees of autism traits. We were particularly interested in studying whether the processing of emotion was influenced by gaze direction (or vice versa), and how this interaction relates with autism trait. Experiment 2 compared two groups of children with and without diagnosis of ASD, matched in age, sex, and cognitive abilities, in an emotion classification task with faces of different expressions and gaze directions, while eye movements were recorded.

## Experiment 1

In Experiment 1 we investigated whether autistic traits in children modulate ERPs related to emotion and gaze processing. Based on the SSH, we studied the interaction between emotional expression and static and dynamic gaze directions. We presented angry and neutral faces with direct and averted gaze, requiring the detection of occasional gaze changes. Based on the findings from the general populations (Latinus et al., 2015), we expected individuals with low autistic traits to show larger N170 amplitudes to faces with averted gaze or changing from direct to averted gaze, compared to the opposite direction. In line with the reported atypical orienting to social stimuli in individuals with ASD (Senju et al., 2005b), we expected this effect to become smaller with higher autistic traits. Moreover, in line with studies using comparable stimulus materials (Senju et al., 2005a; Akechi et al., 2010; Tye et al., 2013, 2014), we expected the gaze effect on the N170 should be stronger for emotional than for neutral faces. Such an interaction of emotion and gaze should diminish with increasing autism trait.

### Methods

#### Participants

Forty-seven Chinese children from the Hong Kong region participated in the study; 16 were excluded because of technical electroencephalography (EEG) issues (*n* = 4), termination of the session prior to completion (*n* = 1), noisy EEG data (*n* = 5), or excessive data loss after EEG preprocessing (*n* = 7), resulting in a final sample of 30 children (19 boys, 11 girls with range 9–12 years, *M*_Age_ = 10; *M*_IQ_ = 100). All children had been tested with the Autism Diagnostic Observation Schedule – Second Edition (ADOS-2; Hus and Lord, 2014; see details below/Min_score_ = 1, *M*_score_ = 3.72, Max_score_ = 12). Both the participant and his/her parent or caretaker signed informed consent, as approved by the institutional ethics review board of the Hong Kong Baptist University.

#### Autism Diagnostic Observation Schedule

The ADOS-2 (Hus and Lord, 2014) is a standardized, semi-structured observational assessment tool used to diagnose ASD and is considered a “gold standard” diagnostic instrument. The ADOS-2 is considered more objective as compared to self-report autistic measures, such as the autism-spectrum quotient (AQ). In particular, the ADOS-2 score is not affected by response biases, individual differences in introspective ability, and honesty of the respondents. The ADOS-2 comprises of four modules designed for different age and language fluency levels. For the present study, Module 4 of the ADOS-2 was used, including the communication and social interaction domains and taking approximately 45 min. The interview was administered and scored by a licensed clinical psychologist according to the diagnostic algorithm outlined in the manual, which can be categorized into non-spectrum, autism spectrum, or autism. In the present study ADOS-2 score was treated as a continuous variable, where a higher score indicates a higher level of autistic trait.

#### Face Stimuli

**FIGURE 1:**
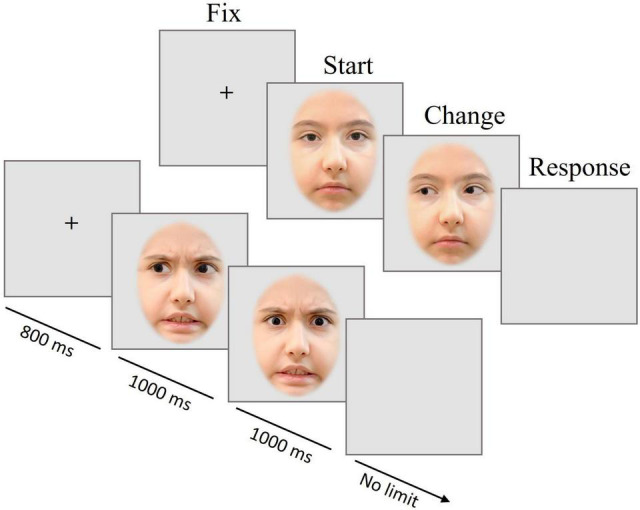
Trial scheme of Experiment 1. Presentation of the fixation cross (FIX) for 800 ms, followed by the first picture with one of two emotional expressions and one of three gaze directions (START) for 1 s, and (in most trials) the gaze change (CHANGE) shown for 1 s; a blank screen prompted a button-press decision, whether a change had occurred or not (RESPONSE). The pictures are for illustration only and were neither taken from the CAFE database nor used in the experiment.

A total of 16 frontal view faces (10 females, 6 males) were selected from the child affective facial expression (CAFE) database (LoBue, 2014; LoBue and Thrasher, 2015) with two different expressions (neutral, angry) and with direct and averted gaze. Gaze direction was photo-edited and the size and position of the faces on the screen was standardized. The eyes were placed at the same horizontal and vertical positions of the screen for every facial picture; and external facial features, such as hair or visible clothing were removed by placing the image into an oval mask. Apparent gaze motion was created from static images by sequentially presenting images with different gaze direction (Figure 1). Therefore, it was important to ascertain that only eye gaze changed between the seamlessly presented pictures with different gaze directions. We manipulated the stimuli as follows: for each individual and emotional expression, the eye region of a picture of the same individual in the data base with averted gaze was copied and carefully pasted into the eye region of the corresponding picture with direct gaze by means of Adobe Photoshop software (version CC 2015, Adobe Systems, San Jose, CA, United States). For each emotion, two gaze changes (from left or right averted to direct gaze and vice versa) and a condition without gaze change were created, with 20% non-change trials in total to prevent expectation effects. Half of all change-trials involved a gaze change from an averted to a direct gaze direction, whereas the other half was a change from direct to averted direction. The emotional type and intensity of the face stimuli were rated by 33 Hong Kong Chinese children aged between 8 and 12 years. The face emotions were correctly identified by 78–100% of the raters.

#### Experimental Design

Figure 1 provides a visualization of the trial structure. Pictures of angry or neutral faces with or without gaze direction change were presented. In most trials, the gaze direction changed after 1000 ms from direct to averted or vice versa. After the disappearance of the second image, a blank screen was shown, during which participants should indicate by pressing a left or right button whether the gaze had changed or not.

#### Electroencephalography Recording

Upon arrival, the parent or caretaker was asked to leave the room during EEG preparation and the experiment. Participants were seated approximately 60 cm away facing an LCD monitor in a dimly lit, sound-attenuated room. The EEG was sampled at a rate of 1000 Hz from 38 Ag/AgCl electrodes mounted in a cap (Waveguard™original) plus one nose reference and connected to an amplifier (eego™mylab, ANT Neuro). Electrode impedances were kept below 20 kΩ using ECI Electro-Gel™. Common reference electrode during recording was CPz. Four additional Kendall™ H124SG ECG electrodes were placed above and below the left eye and at the outer side of each eye to record eye movement.

#### Data Analysis

Participants’ responses were recorded by EPrime software (version 2.0). Mean accuracy data of each participant and condition were analyzed. Overall response accuracy in the change detection task was high with a mean of 96.81% (SD = 0.03) correct responses. No participant gave less than 92.82% correct responses. Response times were not included in the analyses because the task was unspeeded. There were a total of 378 trials per participant. Each trial consists of two intervals, START (the initial face presentation) and CHANGE (gaze change). In the change interval, those cases without changes were dropped from the analysis, leaving 210 trials for START interval and 168 trials for the CHANGE interval. To examine ERP effects of direct eye gaze compared to gaze aversion, the ERPs in the START interval were pooled for the gaze conditions left averted and right averted. Thus, there were just two gaze categories per interval: direct and averted. As a result, for START intervals, each emotion condition (i.e., neutral and angry) had 49 direct trials and 56 averted trials. For the CHANGE intervals, we pooled direct to left averted and direct to right averted trials plus pooling left-averted to direct and right-averted to direct trials, yielding 42 trials for each combination of gaze direction and emotion.

Electroencephalography data were preprocessed in MATLAB R2019a (The MathWorks Inc., Natick, MA, United States) and EEGlab v14.1.1b (Delorme and Makeig, 2004). High- and low-pass filters were set to 0.02 and 30 Hz, respectively. Continuous data was re-calculated to average reference and cut into 1.4-s epochs, including a 100 ms pre-stimulus segment, used for baseline corrections from 50 ms to stimulus onset. On average, 75.2% (*M* = 286.0 intervals out of 378 total intervals, SD = 44.8) of all epochs per participant remained for analysis (START: 128 epochs; CHANGE: 158 epochs). Epochs were removed if they contained extreme values exceeding ±80 μV in any channel. A total of 3.4% of all epochs was excluded because voltages in at least one channel had exceeded ±100 μV (START: 2.5%; CHANGE: 4.4%). ICA was used for eye artifact correction. In total, 12.2% of all epochs was excluded because of eye-movement artifact removal by ICA.

Electrodes and regions of interest (ROIs) were chosen in line with the literature but generally also confirmed in the present data. The electrodes chosen for N170 analysis were P7 and P8 in line with sites of large eye gaze effects (e.g., Latinus et al., 2015). For each condition and participant, average ERPs were generated for epochs synchronized to face onsets and to gaze changes. First, for detecting the N170 amplitude, the minimum voltage was identified in a broader time window from 150 to 300 ms to stimulus onset and after stimulus gaze change. Two distinct time windows (150–190 and 220–270 ms) were extracted from this broad window to make the N170 amplitude easier to observe and score within individuals. Next, the ERP peak amplitude at its latency was measured.

For the EPN, a region of interest (ROIs) was defined according to the literature (Rellecke et al., 2011; Bublatzky et al., 2017): P8, PO8, O2, Oz, O1, PO7, P7, PO5, PO6, PO3, PO4. The averaged EPN amplitude across these electrodes was quantified as mean amplitude in the time windows 200–250, 250–300, 300–350, and 350–400 ms after stimulus onset, separately for the START and CHANGE intervals.

#### Statistical Analysis

Statistical analyses of ERP peak amplitudes and topographies were performed with MATLAB R2019a and the R Software for Statistical Computing (Version 3.2.2). Analyses of variance (ANOVA) were performed on ERP amplitudes with repeated measure on factors Gaze direction (averted, direct) and Emotion (neutral, angry), separately for the START and CHANGE intervals. The sphericity assumption was assessed using Mauchly’s test and adjustments were made applying Huynh–Feldt correction, if needed. Pairwise comparisons were performed between emotional categories, adapting *p*-values according to the Bonferroni correction method.

### Results

**FIGURE 2:**
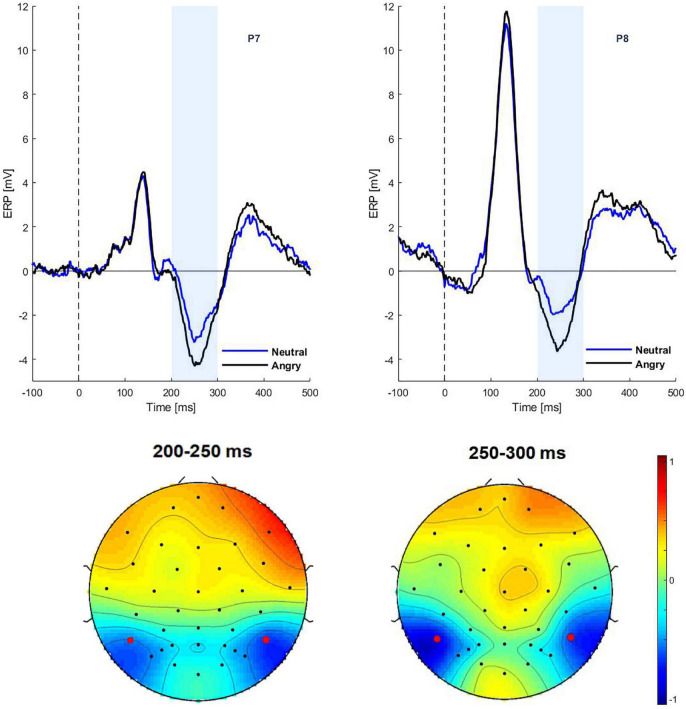
Grand average ERPs in the START interval. *Top*: ERPs at electrodes P7 and P8 for neutral and angry expressions. *Bottom*: Scalp topographies of the emotion effect (angry minus neutral) for the time window of 200–250 and 250–300 ms.

The ERPs to the initial face presentations (START interval) showed a significant effect of emotion on N170 amplitudes during the 220–270 ms interval [*F*(1,29) = 22.28, *p* = 0.02, η^2^ = 0.291], with larger amplitudes to anger (P7: −4.27 and P8: −3.57) than neutral expressions (P7: −3.23 and P8: −1.89) (see Figure 2). There was no effect of gaze direction for the N170 component [*F*(1,29) = 1.95, *p* = 0.07], nor was there an interaction between emotion and gaze [*F*(1,29) = 0.01, *p* = 0.09].

For the EPN component, an emotion effect was observed in the 200–250 ms time window [*F*(1,29) = 6.17, *p* = 0.01, η^2^ = 0.054]. Thus, EPN amplitudes in the angry condition were more negative than in the neutral condition (−1.42 vs. −0.87 μV). There was no effect of gaze direction for the EPN, nor was there an interaction between emotion and gaze [*F*(1,29) = 0.01, *p* = 0.09]. There was no correlation between the Autism Diagnostic Observation Schedule (ADOS) score and any ERP parameter during the start interval.

**FIGURE 3:**
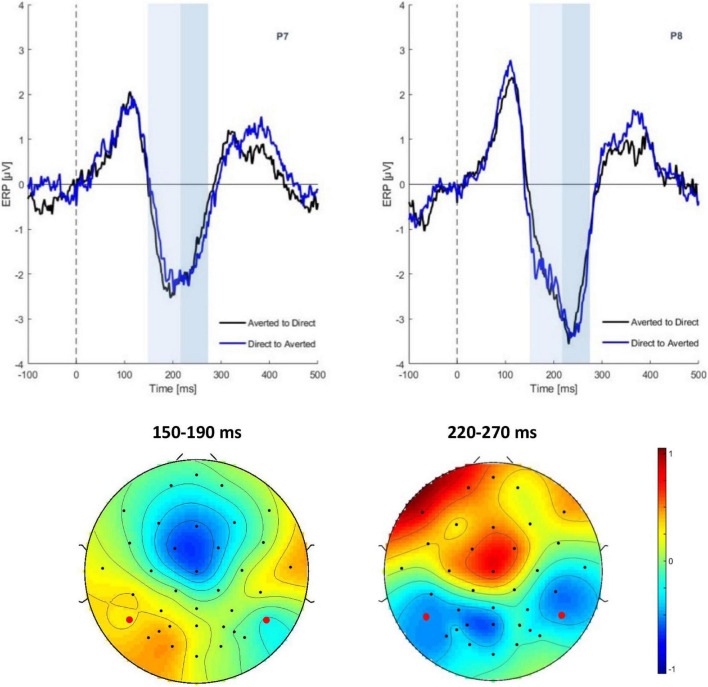
Grand average ERPs in the CHANGE interval. *Top*: ERP waveform of gaze conditions at electrode P7, P8. *Bottom*: Scalp topographies of the gaze effect (ERPs in the direct to averted condition minus the averted to direct condition) in the time segments 150–190 ms (ns) and 220–270 ms.

In the following CHANGE interval, there was a main effect of gaze change on N170 amplitudes at the time window of 220–270 ms [*F*(1,29) = 5.48, *p* = 0.02, η^2^ = 0.0581]. The ERP was more negative for averted than for direct gaze (−3.63 vs. −3.18 μV) (see Figure 3). No significant effect was found on the N170 amplitudes in time window of 150–190 ms [*F*(1,29) = 0.01, *p* = 0.08]. There was neither main effect of emotion nor interaction.

For the EPN ROI there were no significant main effects or interactions in any of the measurement intervals.

**FIGURE 4:**
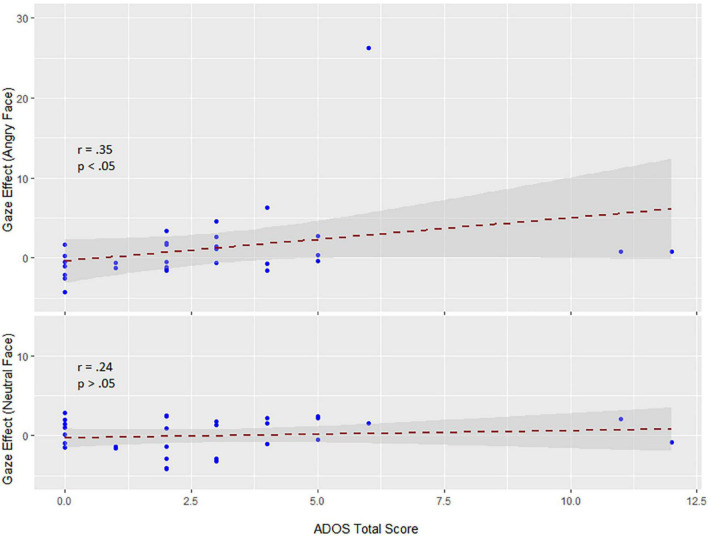
Correlations between the sum sore of the ADOS scale and the gaze effect to angry (top) and neutral faces (bottom) (data are untransformed; correlation is Spearman). Exclusion of extreme values neither changed the positive slope nor the significance of the correlation (*r* = 0.32; *p* < 0.05).

Within the early interval (150–190 ms) of N170 in change interval, there was a significant correlation (Spearman rank-order correlation) of ADOS and the individual gaze change effect for angry faces (ERPs in the direct to averted condition minus the averted to direct condition): *r* = 0.35; *p* < 0.05 (vs. neutral *r* = 0.24; *p* > 0.05) (see Figure 4). The positive correlation indicates that participants with low ADOS scores tended to show the commonly observed larger N170 amplitudes to dynamic gaze changes from direct to averted than for averted to direct. As ADOS scores increased, the gaze effect on the N170 diminished, yielding a positive relationship.

### Discussion

In Experiment 1 we investigated whether ERPs associated with emotion and gaze processing are related to the degree of autistic trait in children. Results concerning the correlation between ADOS scores and ERPs indicate that gaze effects to angry faces and each sub-score of the ADOS (communication and reciprocal social interaction) and the total score were positively correlated, albeit modestly. This correlation across participants was observed in the absence of a gaze effect on the group mean.

Similar to previous reports (e.g., Batty et al., 2011), children in our study showed a very large right-lateralized P1 to the onset of visual stimuli (in the START interval; see Figure 2). Therefore, any experimental effects on the N170 during the START interval were superimposed by the P1, possibly pushing N170 latency toward larger values or just obscuring this component. A gaze effect was only seen in the change interval on the N170 component between 220 and 270 ms at electrodes P7 and P8. This gaze effect followed the typical pattern observed in non-social tasks: gaze aversion (gaze averting to the left or right from direct) elicits a larger negativity than gaze moving from averted to direct.

de Jong et al. (2008) and Akechi et al. (2010) also found differences between ASD and TD groups at P1 and N170. They showed that in ASD individuals, integrating emotional facial expressions and gaze direction is impaired at the level of visual analysis. Nevertheless, we observed a strong early effect of emotion on the N170 in the START interval, which was followed by a typical EPN effect. There were no emotion effects in the CHANGE interval, which is not surprising because in this condition only gaze direction but not the facial expression changed.

Early emotion effects have been reported in a number of studies with adults (Faja et al., 2016). Rellecke et al. (2013) suggested that the N170 may be overlapped by early onset EPN signals. Since only one study (Faja et al., 2016) measured N170 and EPN components simultaneously, it is difficult to tell how far the effects of facial expressions found in the N170 component were driven by overlapping EPN effects (Rellecke et al., 2013). In Faja et al. (2016), fearful facial expressions elicited larger N170 amplitudes than neutral expressions, whereas the EPN was larger to neutral as compared to fearful faces in both ASD and TD groups. It was therefore argued that there is a genuine emotion effect on N170 amplitude. Only Vlamings et al. (2010) and Tye et al. (2014) found main effects of emotion on N170 latency in ASD individuals. Of note, most of the studies reported a main effect of emotion and emotion by group interactions for amplitude and latencies during the processing of fearful and neutral facial expressions. Other emotional expressions have been neglected so far. Some studies reported a main effect of facial emotions only for the control groups, but not for the ASD groups (see Monteiro et al., 2017 for a review). In summary, studies generally indicate differences between ASD and TD individuals in the discrimination of emotional facial expressions, which may thus be a differential characteristic of ASD.

## Experiment 2

While in Experiment 1 emotional expression was not task-relevant but an implicit variable, Experiment 2 required explicit classification of emotional facial expressions, with gaze direction being implicitly manipulated. Eye movements in two closely matched groups of children diagnosed with ASD and TD children were recorded.

According to numerous reports (Kuusikko et al., 2009; Uljarevic and Hamilton, 2012; Lozier et al., 2014), autistic individuals perform worse in recognizing emotional facial expressions than healthy controls, especially in regard to negative emotions such as fear, sadness (Pelphrey et al., 2005; Ashwin et al., 2006; Wallace et al., 2008; Tell et al., 2014), and anger (Rump et al., 2009; Law Smith et al., 2010; Tanaka et al., 2012; Lozier et al., 2014). However, the pattern of gaze direction of the expressor face in combination with positive or negative facial expressions has not yet been extensively studied. Therefore, the task in Experiment 2 required the classification of facial emotions. In order to avoid ceiling effects, we used composite faces where two different emotions were shown in the top and bottom halves, one of which was to be classified. All faces were presented either with direct or averted gaze. Following the assumptions of the SSH, we expected that classification performance in approach-oriented emotions (e.g., happiness and anger) would be better when gaze was direct and in avoidance-oriented emotions (e.g., disgust and fear) when gaze was averted. This effect was expected to be diminished in autistic individuals.

In addition to measuring classification accuracy, we tracked the eye gaze behavior of the participants. If the relevant face half was at the bottom, we expected eye movements to be reflexively attracted to the eyes. This effect was assumed to be less pronounced in the ASD group. If the top half of the face was relevant, the particular emotion was expressed mostly around the eye region, that is, fixation on the eyes should be helpful for task performance. If individuals with ASD tend to avoid eye contact, we expected them to fixate less on the eyes than normal controls, especially in expressions with direct gaze.

### Methods

#### Participants

By applying propensity score matching (Austin, 2011), 22 German-speaking TD children were matched by age (8–18 years), sex and intelligence with 22 children with a diagnosis of ASD (8 females and 14 males). The clinical diagnosis of ASD (DSM-V) was given or confirmed by an expert adolescent psychiatrist and substantiated by reviewing the medical files of the individuals in addition to the available diagnostic documents. More than half of the ASD sample received extensive clinical training targeting social competencies prior to study participation. In addition, subgroup of (*n* = 9) autistic children were reported by the clinician to be hitherto poorly trained with respect to social competence. The study was conducted in according to the Declaration of Helsinki and it was approved by the Ethics Committee of the University of Greifswald.

#### Stimuli

**FIGURE 5:**
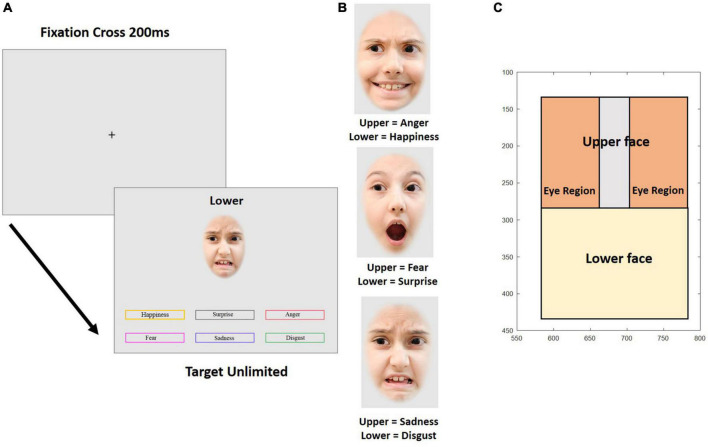
Experiment 2. **(A)** Temporal sequence of a trial in the emotion composite task. Example trial with the target emotion “disgust” at the bottom half of the face. **(B)** Examples of emotional composite faces. **(C)** Parameterization of the variables for gaze behavior. The axes show pixels coordinates on the screen. The pictures are for illustration only and were neither taken from the CAFE database nor used in the experiment.

Stimuli were taken from the child affective facial expression (CAFE) database (LoBue, 2014; LoBue and Thrasher, 2015) consisting in 48 images of 8 different identities. Faces of four girls and four boys (between the ages of 4.6 and 6.8 years) displaying expressions of six basic emotions were selected according to the accuracy of the expression. The images were modified and optimized for the experimental design (see Figure 5) using Adobe Photoshop CS6 2012 (by Adobe Systems and the Adobe Photoshop development team © 1988–2016, Version 3.0 × 64).

The external features of the faces, such as ears and hairline were removed by overlaying an elliptical mask. Then, the faces were horizontally divided at the middle of the bridge of the nose. Thus, half-faces of each emotional expression and each individual picture were prepared for recombination. Face halves were reassembled within a given identity according to a composite design scheme. Nine different re-combinations per identity were created, yielding a total of 72 composite faces. Upper face halves showed fear, sadness, or anger, emotions that are most easily recognized in the top part of the faces and lower face-halves showed happy, surprise, or disgust, emotions that are best recognizable from the lower face (see Figure 5). The separation line between the face halves was always visible. Each composite face was 200 × 300 pixels in size. Figure 5B shows examples for composite faces of a female identity. Finally, 36 composite faces were edited to change gaze direction (18 faces each displaying left and right averted gaze) while 36 faces showed direct gaze.

#### Experimental Design

The emotional composite face task validly and reliably measures the ability to recognize emotion expression (e.g., Wilhelm et al., 2014; Hildebrandt et al., 2015). Figure 5A shows an example for one trial. Each trial began with a fixation cross presented for 200 ms in the middle of the screen, followed by a composite face together with six color-framed labels for the six emotions and a prompt (“TOP” or “BOTTOM”) placed above the composite face, cueing which face half was to be categorized. Emotion labels and the face remained on the screen until a decision was made about the displayed emotion by clicking one of the emotion labels with the mouse. The task started with nine practice trials, where participants were given feedback about the correctness of their response. In the following experimental trials, no feedback was provided. In total, 72 experimental trials were presented in random order.

#### Eye Tracking

The gaze behavior of participants was tracked with a remote device [*Eye Tribe Tracker* (from *The Eye Tribe ApS* © 2013–2016)], recording binocular fixation positions in 60 Hz mode using an integrated camera. The eye tracker was placed below the monitor aiming at the eye region of the participant. Prior to the task, the device was calibrated twice by instructing the participants to follow the movements of a sphere across the screen with their eyes. If this calibration process was completed with satisfactory quality (at least three out of five “stars”), the experiment started. If necessary, participants were given feedback about the tracking quality on the screen, allowing to correct their sitting position, direction of view or posture. The distance to the eye tracker was individually adjusted to achieve the best possible measurement quality. During the experiment, participants were not to move their heads, but keep their eyes on the screen. The raw data of the eye tracker were converted into fixation points on the screen surface using a coordinate system. The resolution of the eye tracking system was 17 ms, which is also the lower bound of the fixation times. Worthy to mention, the eye tracking data was analyzed based on all eye gaze position at every time point.

Eye tracking behavior was analyzed in two ways. Firstly, the mean of the median vertical position on the screen was calculated for 10 consecutive intervals of 200 ms for a total of 2 s from stimulus onset. This was done for each combination of emotional expression, gaze direction of the face on display, and participant. Since the vertical position in the picture does not allow to address the question whether the eyes were directly fixated when participants looked at the upper half of the faces, we conducted an additional more fine-grained analysis of the fixation behavior in the upper face half. Three regions were defined in the upper face half, representing each eye and the area in between (see Figure 5C). Then, we determined the total fixation duration in each of these regions during four consecutive intervals of 500 ms after stimulus onset. From these fixation durations we calculated an eye avoidance index (EAI), reflecting the relative amount of time spent outside as compared to inside the eye regions


$$\text{EAI}=\left({\text{FD}}_{\text{inter}-\text{eye}}-{\text{FD}}_{\mathrm{left}\mathrm{eye}}+{\text{FD}}_{\mathrm{right}\mathrm{eye}}\right)/{\text{FD}}_{\mathrm{upper}\mathrm{face}}$$

where FD is the fixation duration, and the indices inter-eye, left eye, right eye, and upper face correspond to the designated face regions (see Figure 5C). The larger EAI, the less time is spent on the eye area if fixation is in the upper face half.

### Results

**FIGURE 6:**
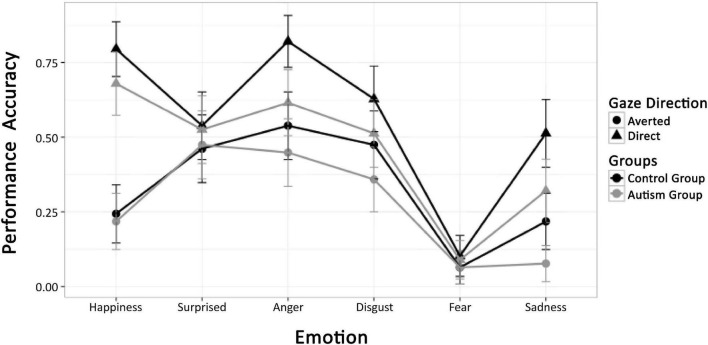
Mean performance accuracy of the ASD and TD groups for all emotion conditions and direct and averted gaze of stimulus faces.

Performance accuracy is shown in Figure 6. Clearly, fear was extremely difficult to classify and surprise processing was not influenced by gaze direction or group at all. We therefore confined all further analyses to the expressions of happiness, anger, disgust, and sadness, orthogonally combing the display in the top and bottom halves of the composite faces (anger and sadness vs. happiness and disgust) and the tendency to approach and avoid (happiness and anger vs. disgust und sadness). ANOVA with factors group and repeated measures on emotion (four levels) and gaze direction yielded a main effect of group, indicating that individuals with ASD showed lower accuracy than TD participants [*F*(1,54) = 4.37, *p* = 0.04, η^2^ = 0.0208]. In addition, main effects of gaze [*F*(1,54) = 7.34, *p* = 0.001, η^2^ = 0.0119] and emotion [*F*(3,162) = 1.39, *p* = 0.001, η^2^ = 0.0901], and an interaction of emotion and gaze [*F*(1,162) = 7.91, *p* = 0.001, η^2^ = 0.0358] were observed. As illustrated in Figure 6, these effects are due to variable performance accuracy across emotions (*M*_anger_ = 0.65; *M*_disgust_ = 0.55; *M*_happy_ = 0.56; *M*_sadness_ = 0.36), better emotion recognition for expressions with direct than averted gaze (*M* = 0.56 vs. 0.37) and the gaze effect depending on emotion, being largest for happiness, intermediate for anger and sadness, and intermediate for disgust. However, there was no significant interaction of group with emotion, gaze, or both factors (*F*s < 1). The ANOVA was repeated for the subgroup of autistic children less trained in social competence. Again, no significant interaction between emotion and group [*F*(1,48) = 0.71, *p* = 0.05] was obtained. All other results were in line with those in the full sample.

**FIGURE 7:**
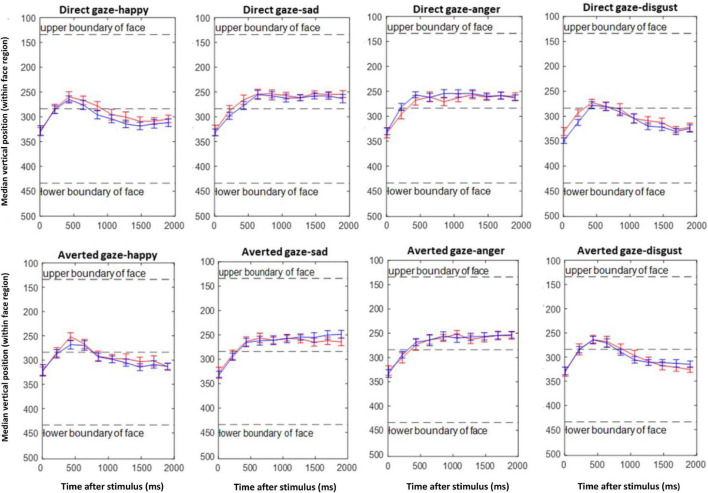
Emotion composite task. Mean of median vertical gaze positions of the ASD (red) and TD (blue) groups during the first 2 s after stimulus presentation in consecutive time bins of 200 ms.

Figure 7 visualizes the gaze behavior within the first 2 s of stimulus presentation. At around 500 ms after stimulus onset, a general tendency to look at the upper half of the faces (into the eye region or at the prompt) can be observed. This is either continued until the end of the recording epoch, or fixation turns toward the lower face half, depending on whether the upper or lower face half was task-relevant (i.e., anger, fear vs. happiness, disgust). In any case, there is no evidence for a differential main effect or interaction of emotion with gaze direction of the stimulus face for the participant groups. This impression was confirmed by ANOVA with factor group, and repeated measures on gaze, for each emotion, which did not show any significant interaction of group and gaze: angry [*F*(1,42) = 0.92, *p* = 0.34], happy [*F*(1,42) = 0.37, *p* = 0.54], disgust [*F*(1,42) = 1.53, *p* = 0.22], and sad [*F*(1,42) = 2.55, *p* = 0.11].

**FIGURE 8:**
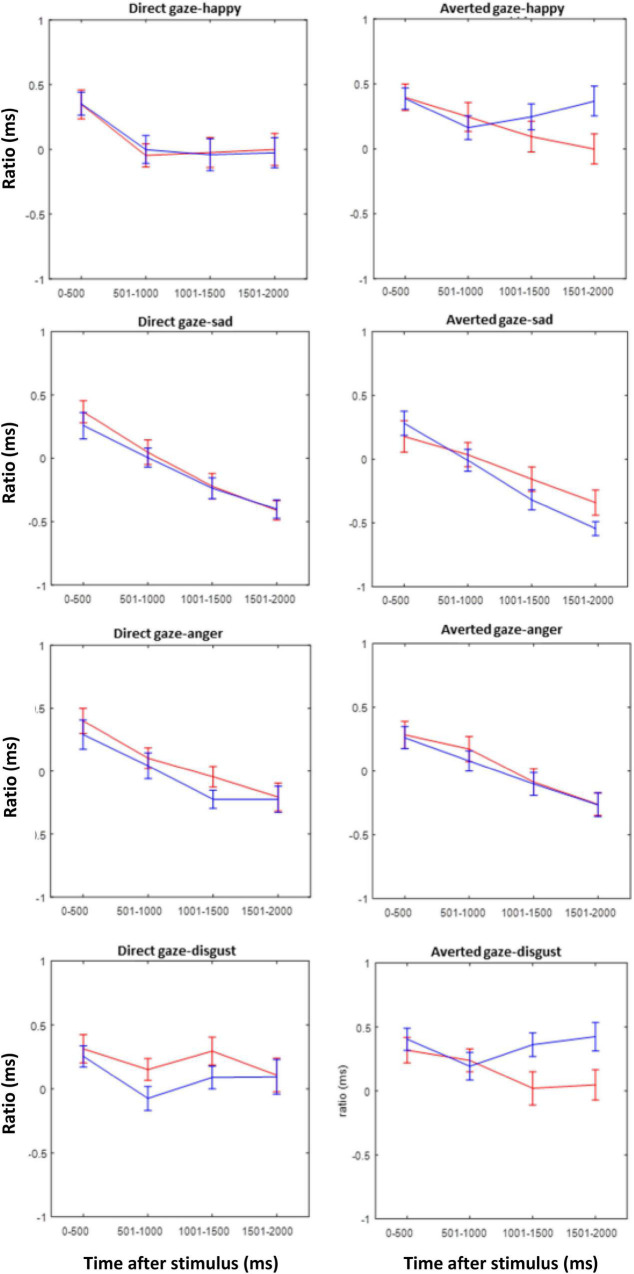
Eye avoidance index (EAI) for the ASD (red) and TD (blue) groups during the first 2 s after stimulus presentation in intervals of 500 ms for all emotions and both gaze directions of the stimulus faces.

Figure 8 shows the EAI for each emotion and gaze direction of the composite face, superimposed for the two groups. The EAI index indicates that except for disgust, there is mostly an overlap between the groups. For disgust, however, the EAI revealed an interaction of group and gaze direction of the stimulus face, as confirmed by the ANOVA of the EAI with factor group and repeated measures on time [*F*(1,42) = 6.99, *p* = 0.01, η^2^ = 0.0256]. *Post hoc* tests showed an effect of gaze direction for TD children [*F*(1,29) = 18.03, *p* = 0.001] who looked more at the eyes when gaze was direct than when it was averted. In contrast, in the ASD group there was no effect of gaze direction [*F*(1,29) = 0.68, *p* = 0.4.09].

### Discussion

In Experiment 2 we investigated the ability to recognize emotions from facial expressions and its modulation by gaze direction in ASD and TD children by means of the emotion composite task. The observed overall lower performance of the ASD group as compared to TD children might reflect a global deficit in categorizing facial expressions as reported in many other studies (Wong et al., 2008; Chronaki, 2016). However, since we had no non-emotional control task, it might also reflect a more general phenomenon in the ASD group, e.g., task compliance related differences between the groups.

Gaze behavior was strongly modulated by the position of the relevant face half. For emotions in the lower half (happiness, disgust) there was a tendency to look at the upper face half after which fixation returned to the lower half. Importantly, this was not modulated by the gaze direction of the picture nor by the participant group. For the emotions displayed in the upper face half, the participants’ fixations remained in this part but again, there was no modulation by picture gaze or group. In contrast with Tanaka and Sung (2016), it is noted that none of the present findings support the active avoidance of eyes in ASD individuals, even in direct gaze conditions, which should have been evident in our EAI.

Nevertheless, a difference between the groups was present when disgust was the relevant emotion to be classified and when the EAI was considered. According to this index, when the relevant (bottom) face half showed a disgusted expression, TD children looked more at the eyes of the composite face when gaze was direct than when it was averted. Since the prompted face half was in the lower part of the composite face, this effect should be considered implicit, maybe a reflexive eye contact even though it was task-irrelevant. In stark contrast, no such effect was present in children diagnosed with ASD. These findings point toward an insensitivity for gaze direction in the ASD group in an emotion, where normal children are highly sensitive to gaze direction. It is of interest that disgust was the only emotion condition, where TD children showed such a gaze sensitivity. Therefore, the present results may not support an emotion specificity of this effect; the effect might well general to other emotions if the tasks were more sensitive.

## General Discussion

To our knowledge, this is the first study reporting on the interaction between facial emotion processing and gaze direction in children with different levels of autistic traits. Our starting point was that according to the SSH, approach-related emotions, for example, happiness and anger, are more easily recognized when the observer is directly looked at. In contrast, avoidance-related emotions, such as sadness and disgust, are better recognized with averted gaze. We expected that these benefits would be less pronounced or even absent in children with ASD as compared to TD children.

The present data provided some limited support of the SSH in its original form. In Experiment 1, the facial emotion expression was implicit and the gaze direction was incidental to the task. Yet, we did not find an interaction between emotion and gaze in the ERPs. In Experiment 2, when participants were required to explicitly categorize emotion expressions, performance was indeed best when gaze was direct. This effect was most pronounced for smiles and anger, which are both considered approach-related emotions. However, the avoidance-related emotion, sadness, revealed a similar effect as anger, and disgust recognition was facilitated by direct as compared to averted gaze, albeit with a relatively small effect.

Although the SSH cannot only partially account for the full pattern of associations revealed by the present data in TD children, we found some evidence that autistic trait is related to diminished sensitivity to gaze in the context of processing facial emotions. Although there was no interaction of gaze direction and emotion at the group level in Experiment 1, the gaze effect in the N170 amplitude elicited by angry faces correlated positively with the ADOS score. This correlation is broadly in line with the SSH, which assumes an interaction between eye gaze and emotion. Thus, when gaze direction (from direct to averted in our experiment) was combined with the intent communicated by a specific expression (anger in our study), the perceptual analysis of that emotion was enhanced. Therefore, the observed correlation between the N170 gaze effect in angry faces and its attenuation with increasing ADOS seems to fit the hypothesis: avoiding/averting gaze is a signal shared with the non-affiliative emotion of anger in neurotypical (low ADOS) individuals. And the loosening or reversal of this association at higher ADOS scores is in line with what one might expect for higher autistic trait expression. Hence, these data are consistent with the observation that in a naturalistic setup, in which dynamic emotional gaze cues require the integration of emotional information and gaze information, individuals with ASD differ from TD individuals in their responses to eye gaze in emotional faces.

Although in Experiment 2 only a global deficit in emotion recognition, independent of the particular emotion and gaze direction was found between in the ASD relative to the TD group, eye tracking data revealed that TD children fixated longer on the eyes when the facial emotion expression was disgust, while the ASD group did not demonstrate such pattern. Again, this would indicate a lack of sensitivity for gaze direction in the ASD group in the context of a specific emotion. Hence, together, our results indicate a partially diminished sensitivity in processing gaze direction in emotional faces among children with ASD or high in autism trait. The relative indifference to gaze direction may be an important problem in understanding emotional expressions where gaze is an important constituent. Therefore, the present findings show that indirect multimodal measures of emotion/gaze processing may be able to uncover subtle deficits of ASD-related traits, where performance is not sensitive enough to indicate such problems. The present findings indicate that it would be promising to increase the spectrum of emotions investigated while combining eye tracking in unrestrained viewing conditions in stimuli with varying gaze behavior. Recent methodological advances, such as the co-registration of EEG and eye movements (Dimigen et al., 2011) and the employment of dynamic stimuli in gaze-contingent display situations (Stephani et al., 2020) already make such an approach feasible.

## Data Availability Statement

The raw data supporting the conclusions of this article will be made available by the authors, without undue reservation.

## Ethics Statement

The studies involving human participants were reviewed and approved by review board of the Hong Kong Baptist University and Ethics Committee of the University of Greifswald. Written informed consent to participate in this study was provided by the participants’ legal guardian/next of kin. Written informed consent was obtained from the individual(s) for the publication of any identifiable images or data included in this article.

## Author Contributions

SB-A: conceptualization, methodology, investigation, formal analysis, and writing – original draft. GL: investigation and writing – review and editing. GO: formal analysis and writing – review and editing. CZ: writing – review and editing. AH: resources, conceptualization, methodology, and writing – review and editing. WS: conceptualization, methodology, writing – original draft, and supervision. ML: conceptualization, methodology, writing – original draft, and resources. All authors contributed to the article and approved the submitted version.

## Conflict of Interest

The authors declare that the research was conducted in the absence of any commercial or financial relationships that could be construed as a potential conflict of interest.

## Publisher’s Note

All claims expressed in this article are solely those of the authors and do not necessarily represent those of their affiliated organizations, or those of the publisher, the editors and the reviewers. Any product that may be evaluated in this article, or claim that may be made by its manufacturer, is not guaranteed or endorsed by the publisher.
